# A Comparative Analysis of Stress Relief, Life Satisfaction, and Quality of Life Based on Marriage Status and Gender Among Members of Society Participating in Exercise

**DOI:** 10.3390/bs15040453

**Published:** 2025-04-01

**Authors:** Ji-Hye Yang, Sicheol Jung, Hyejin Yang, Chulhwan Choi, Chul-Ho Bum

**Affiliations:** 1 Department of Human Movement Studies and Special Education, Old Dominion University, Norfolk, VA 23529, USA; jyang009@odu.edu; 2 Department of Physical Education, Graduate School, Kyung Hee University, Seocheon-dong 1, Giheung-gu, Yongin-si 17104, Gyeonggi-do, Republic of Korea; jsc6277@naver.com (S.J.); y0108577@khu.ac.kr (H.Y.); 3 Department of Physical Education, Gachon University, 1342 Seongnam-daero, Sujeong-gu, Seongnam-si 13120, Gyeonggi-do, Republic of Korea; 4 Department of Golf Industry, College of Physical Education, Kyung Hee University, Seocheon-dong 1, Giheung-gu, Yongin-si 17104, Gyeonggi-do, Republic of Korea

**Keywords:** stress relief, life satisfaction, quality of life, members of society, exercise

## Abstract

Modern members of society tend to feel stressed in their workplace and at home, but exercising has been shown to effectively reduce stress and increase quality of life and satisfaction. This study aimed to investigate and compare stress relief, life satisfaction, and quality of life based on marriage status and gender among members of society who participate in exercise. We used a questionnaire survey with 311 participants, and the data were analyzed using descriptive statistics, validity and reliability, a multivariate analysis of variance (MANOVA), and a post hoc analysis. The study results showed that the single groups demonstrated higher mean scores in stress relief than the married groups. Conversely, the married groups showed higher mean scores in life satisfaction and quality of life compared with the single groups, and there were no gender differences in any of the results. In conclusion, it seems that these results were affected by the single groups’ high autonomy and the married groups’ sense of stability in the family.

## 1. Introduction

The era of ubiquitous systems emerged as a result of modern technology, and life today has become more prosperous and convenient because of them. However, Yoon et al. (2010) suggested that stress continuously occurs in people’s social relationships. This is because, in the case of members of society, feeling the high pressure and responsibility of work, anxiety to survive while fiercely competing, and pressure to achieve a promotion cause stress. These weak points of social construction remain, and, specifically, bad communication in the workplace causes discord or even creates emotional battles (Y.-K. Lee, 2004). Moreover, Beck et al. (1993) and Reynolds (1998) highlighted that if people encounter these problems persistently, they may ruminate excessively about them and it can cause suicidal thoughts.

These stresses are also observed in various home environments. Y.-S. Park et al. (2017) found that parents experience stress due to the pressure and burdens related to raising and educating their children. Furthermore, parents’ extreme interest in education causes their children to feel high pressure (Y. S. Park & Kim, 2006). For example, parents who think their children’s low educational results are their fault lead to interference, causing negative emotional problems in both parents and their children (U. Kim & Park, 2006). Furthermore, people who have recently been married and do not have children also experience similar stress (e.g., conflict with a partner) in the home environment (Neff & Broady, 2011). Adamczyk (2017) investigated young single people and identified their stress as manifesting in anxiety, depression, and romantic loneliness. Similarly, modern members of society experience high stress in both the workplace and home environments regardless of their marriage status.

However, persistent physical activity may contribute to reducing these problems. According to Biswas et al.’s (2015) study, the authors demonstrated that adults who continuously perform physical activity care about their bodies, reduce their stress through positive thinking, and develop a healthy life. In addition, performing physical activity helps people to recognize a relationship’s importance in home environments (Cobb, 1976), communicate well in social relationships (J.-R. Lee, 2020), and effectively improve their quality of life (Wankel & Kreisel, 1985). Furthermore, performing a high difficulty level of systematic physical activity helps people to judge themselves intuitively and not have preconceived notions about other people (Gruber, 1986), which is very helpful in creating good relationships.

Similarly, it seems like physical activity can positively change most parts of human life. To prove this, in Gruber’s (1986) meta-analysis study, the author found that adults who frequently perform physical activity have positive and appropriate personality traits. According to Collingwood’s (1972) study, adults who performed high-frequency physical activity were more likely to show a good effect on emotional aspects than adults who performed lower-frequency physical activity. R. J. Sonstroem (1984) suggested that physical strength improvement and positive physical enhancement can develop individuals’ quality of life, and J.-H. Lee (1999) also emphasized the importance of interest in physical activity. Therefore, this study focused on members of society who are single or married to investigate whether physical activity has positive effects on their psychological factors and whether there are significant differences in terms of gender or marital status. Finally, it is an undeniable fact that there are very few comparable previous studies, so this proves this study’s importance and value. This study will provide basic data that help to identify the differences in people’s psychological factors who work in society and participate in exercise. The hypothesis proposed in this study is as follows:

**Hypothesis 1.**  *There will be differences in stress relief, life satisfaction, and quality of life among four groups (single males, single females, married males, and married females).*

### Literature Review

Human life has become productive and convenient due to economic growth. Nevertheless, Cole et al. (2006) mentioned that a continuous working life makes people’s emotional aspects negative and more serious, reduces motivation due to stress, and increases a sense of depression. Furthermore, modern people are negatively affected by societal relationships that are considered important in the process of life, specifically causing psychological distress (Bartholomew & Horowitz, 1991). These problems of daily personal stress and work stress have become a serious problem (Rich et al., 1991), and Langlois and Morrison (2002) noticed that this can cause the emotional aspect to deteriorate, and in severe cases, this can lead to suicidal behavior. However, cases of dangerous stress levels can be reduced through regular physical activity and it can also change negative physical and emotional aspects to positive ones and increase happiness by reducing stress (Bize et al., 2007; Löllgen et al., 2009). Moreover, the significant impact of physical activity on adults’ stress relief, life satisfaction, and quality of life has been proven in many previous studies (e.g., An et al., 2020; Erdoğan Yüce & Muz, 2020; M. Park et al., 2020). Despite these general findings on stress, little attention has been given to how stress levels and coping mechanisms differ between married and unmarried individuals. Marriage may provide emotional and social support that alleviates stress, while unmarried individuals may experience different sources and intensities of stress due to the absence of such support structures. This distinction is essential to understanding variations in emotional well-being and quality of life between these groups.

Modern society has changed due to rapid economic development, which has changed people’s lifestyles. However, in the case of life satisfaction, individuals usually want to gain satisfaction from others’ trust, not themselves. Also, there is a tendency to be possessive of others to continue to gain satisfaction (Clarkson, 1992). Sometimes, this tendency is related to an obsession with relationships, which causes physical and emotional stress, and has negative impacts on daily life (Ivanchevich & Matteson, 1980; Mason, 1975). These problems can be seen in home environments. For example, some parents exhibit controlling behaviors in order to shape their children into an ideal form (Clemens & Axelson, 1985), and weak communication in husband-and-wife relationships can cause reduced life satisfaction (Pleck, 1977). However, people can resolve these problems and change their view on life more positively through physical activity (Ragheb & Griffith, 1982). Additionally, I.-S. Park and Kim (2011), Pender (1987), and R. J. Sonstroem (1978) highlighted continuous physical activity because it can enhance not only physical but also psychological factors, which can change the perception of life positively. Similarly, physical activity is a very important factor in individuals’ life satisfaction and their overall quality of life.

Today, people experience a new turning point in adult life as they take on work and family responsibilities. However, Kocalevent et al. (2013) mentioned that if people cannot adapt to these environmental changes, they will feel lethargy and their quality of life will be reduced. Furthermore, the main problem is that they show bad eating habits (e.g., excessive intake of food or alcohol) to release their stress (Peirce et al., 1994; Russel et al., 1999). Eventually, these bad eating habits cause physical problems such as symptoms of dependence on intake and obesity (Caroline et al., 2020), and an unhealthy body aggravates one’s mental health, which reduces the quality of life (Ross et al., 2021). Even though people may face many serious problems, they can maintain their physical and mental health and high quality of life through physical activity (Gregg et al., 2003). Moreover, physical activity has other positive effects like reducing stress, increasing self-love and self-esteem, and feelings of high self-value (Morgan & Goldston, 1987). Also, R. Sonstroem and Morgan (1989) mentioned that people can feel the preciousness of all things and improve their sense of respect for others with increasing quality of life.

In the case of married individuals, physical activity enhances marital relationships through shared exercise (Yorgason et al., 2018) and can influence depressive symptoms through a partner effect (Monin et al., 2015). On the other hand, M. Kim and Lee’s (2024) study found that single females had a higher mean score for exercise continuation, which involved having friends to exercise with and developing exercise skills, compared to that of married females. This suggests that exercise helps single women expand their social relationships and improve their physical abilities.

Previous studies have primarily focused on exercise duration and intensity based on marital status or gender (e.g., Hull et al., 2010; Nomaguchi & Bianchi, 2004; Rapp & Schneider, 2013) or have examined the relationship between physical activity and psychological factors among workers (Arslan et al., 2019; Biernat et al., 2010; Lindberg et al., 2018). However, to our knowledge, no published research has specifically explored differences in psychological factors based on marital status and gender among individuals who participate in physical activity. To address this research gap, this study aims to examine how stress relief, life satisfaction, and quality of life vary based on marital status and gender among physically active individuals, offering new insights into the relationship between exercise, mental well-being, and social dynamics.

## 2. Materials and Methods

### 2.1. Data Collection

This study investigated the mean differences in stress relief (physical, mental, and social), life satisfaction, and quality of life among four groups divided according to sex and marital status to verify the possibility that physical exercise could have a positive impact on mental health. Study participants were limited to adults over the age of 20 who regularly participated in exercise in the Republic of Korea. To collect data, on/offline survey questionnaires (a quantitative research design) were distributed to survey respondents who voluntarily participated in this study. The online participants who had been personally contacted used an online link (Naver survey platform) to conduct the survey. The study purpose and instructions for study participation were disclosed in advance. The data collection took place for four months starting in August 2024 based on the convenience sampling technique. A convenience sampling method was employed due to practical constraints such as accessibility and time. While this method may introduce sampling bias and limit generalizability, we attempted to mitigate this by ensuring a diverse demographic composition (age, gender, and exercise frequency) that reflects the broader population of adults engaging in exercise in Korea. We acknowledge this limitation and recommend that future studies consider alternative sampling strategies such as stratified or quota sampling to enhance representativeness.

### 2.2. Instrument

This study measured stress relief of survey respondents via questionnaires modified by Y. G. Lee (2019), who investigated effects of the enjoyment factor in sports on stress relief. The stress factor consists of three sub-factors: (a) physical (three items), (b) mental (three items), and (c) social (three items). The items utilized a 5-point Likert scale ranging from 1 (“not at all”) to 5 (“very much”). Next, the factor used to understand the life satisfaction as a single factor through participating in exercise was applied to questionnaires (four items) modified by Kwon (2013), exploring effects of regular exercise participation on psychological status such as happiness, fun, and life satisfaction. The items utilized a 5-point Likert scale ranging from 1 (“not at all”) to 5 (“very much”). Lastly, the factor of quality of life is also a single factor (five items) modified by Jun (2021), exploring the impact of mental health on quality of life in middle-aged men. The items utilized a 7-point Likert scale ranging from 1 (“not at all”) to 7 (“very much”). In this study, these measures showed acceptable validity in an exploratory factor analysis (EFA): stress relief (range 0.746 to 0.886), life satisfaction (range 0.813 to 0.910), and quality of life (range 0.814 to 0.938).

### 2.3. Data Analysis

The data collected through the survey were statistically analyzed using SPSS version 28.0. First, sociodemographic data via descriptive statistics were analyzed. Second, scale validity was examined using an EFA. Third, scale reliability was examined applying Cronbach’s alpha coefficients. Finally, multivariate analysis of variance (MANOVA) and a post hoc analysis were implemented to find statistically significant mean differences in five dependent variables among the four groups (e.g., single males, single females, married males, and married females). The MANOVA for comparative study design in social sciences was utilized as a statistical method with more than two groups and more than two variables (Choi et al., 2024, 2023; D. K. Kim et al., 2023; Yang et al., 2024).

## 3. Results

### 3.1. Survey Respondents

Through the data collection process, this study obtained 311 survey questionnaires. To assess the effect size for group comparisons in the statistical analysis, the G*Power 3.1.9.7 program was utilized. For the F test analysis (MANOVA), the following settings were applied: the effect size was set at 0.0625 and alpha error was 0.05, with three groups and five dependent variables. The results indicated that a sample size of at least 201 participants is necessary to sufficiently capture the specified effect size. Therefore, this study meets the required sample size to ensure adequate power for detecting the intended effects, thereby reinforcing the validity of the findings.

Through descriptive statistics, sociodemographic information (e.g., sex, age, marital status, education, employment status, and frequency of participating exercise per a week) was analyzed from the survey respondents. In addition, sex and marital status were applied as independent variables to segment study participants for the comparative analysis in this study. Based on the collected independent variable information, the survey respondents were segmented into four groups: group 1, single males; group 2, single females; group 3, married males; and group 4, married females. Detailed descriptive statistics of the survey respondents in the four groups are presented in Table 1.

### 3.2. Scale Validity and Reliability

The EFAs were conducted using Principal Component Analysis (PCA) with Varimax rotation to examine the underlying structure of the variables. A PCA was selected because the primary goal was to reduce dimensionality and summarize the data structure, rather than to identify latent constructs. The variables analyzed included stress relief: (a) physical (four items), (b) mental (four items), and (c) social (four items); life satisfaction (four items); and quality of life (five items).

From the factor structure of stress relief, the Kaiser–Meyer–Olkin test found sample adequacy (0.878). In addition, Bartlett’s test of sphericity met statistical significance (χ^2^ = 2874.007, *df* = 66, *p* < 0.001). The three retained sub-factors of stress relief (physical, mental, and social) accounted for 78.175% of the total variance. Also, these had satisfactory eigenvalues and factor structure coefficients that ranged from 0.746 to 0.886.

Next, from the factor structure of life satisfaction, the Kaiser–Meyer–Olkin test found sample adequacy (0.829). In addition, Bartlett’s test of sphericity met statistical significance (χ^2^ = 832.732, *df* = 6, *p* < 0.001). The original tool consists of four items related to life satisfaction. The initial factor analysis verified an intact single factor (range 0.813 to 0.910) without excluding any items.

Last, from the factor structure of quality of life, the Kaiser–Meyer–Olkin test found sample adequacy (0.896). In addition, Bartlett’s test of sphericity met statistical significance (χ^2^ = 1546.730, *df* = 10, *p* < 0.001). The original tool consisted of five items related to life satisfaction. The initial factor analysis verified an intact single factor (range 0.814 to 0.938) without excluding any items.

For the internal consistency (scale reliability), all five dependent variables had acceptable (greater than 0.70) Cronbach’s alpha coefficients (Nunnally & Bernstein, 1994): mental (α = 0. 920), social (α = 0.920), and physical (α = 0.865) stress relief, life satisfaction (α = 0.894), and quality of life (α = 0.945). Table 2, Table 3 and Table 4 present the validity and reliability results for stress relief, life satisfaction, and quality of life, and the factor loadings of each sub-factor were highlighted in bold.

### 3.3. Multivariate Analysis of Variance

The MANOVA was implemented in order to verify statistically significant mean differences in five dependent variables. First, the homogeneity of covariance was tested (Box’s *M* = 111.047, *F* = 2.395, *p* < 0.01). Second, Levene’s Test of Equality of Error Variances was performed for the assumption of homogeneity of variances for each dependent variable. The results revealed that, while three dependent variables (physical stress relief, life satisfaction, and quality of life) were statistically significant (<0.05), indicating equal variance across the levels of independent variables, the other variables, mental (*p* = 0.057) and social (*p* = 0.128) stress relief, were not statistically significant. While the results of Levene’s test partially supported the assumption of homogeneity of variance, Stevens (2009) emphasized the robustness of the F statistic when the sample size is sufficiently large and the differences in sample sizes across groups are not substantial. Based on this rationale, the researcher proceeded with the analysis. Third, statistically significant mean differences among four age groups were found (Wilks’ Lambda = 0.503, *F* = 15.768, *p* < 0.01, *η*^2^ = 0.205). Specifically, from this MANOVA, there were statistically significant mean differences in all of the dependent variables. Additionally, to determine which of the four groups revealed statistically significant mean differences, a Scheffe post hoc analysis was performed. As a result, the effect size (*η*^2^) of all variables showed medium to large effect sizes (0.100 to 0.241) based on Cohen’s (1988) criteria (small = 0.01, medium = 0.06, large = 0.14). On three sub-factors, (a) physical, (b) mental, and (c) social stress relief, the two unmarried groups, single males (group 1) and single females (group 2), showed higher mean scores than the other two groups (married males, group 3, and married females, group 4). On the other hand, for the factors of life satisfaction and quality of life, this study found higher mean scores in the two married groups, the married males (group 3) and married females (group 4), than in the other two groups (single males, group 1 and single females, group 2). The detailed results of the MANOVA and post hoc analysis are reported in Table 5 and Table 6.

## 4. Discussion

The goal of this study was to investigate the differences in stress relief, life satisfaction, and quality of life among society members who participate in exercise based on their marriage status and gender. The results showed that the participants in the four groups showed differences in all variables (physical, mental, and social stress relief, life satisfaction, and quality of life). Furthermore, these results seemed to be more affected by marriage status than gender.

In detail, in the case of stress relief, which includes three sub-variables (physical, mental, and social), the single males (group 1) and single females (group 2) showed higher mean scores than the other two married groups. These results seem to be affected by single people’s characteristics, in that they have more leisure time than people who live with family (Y. G. Lee & Bhargava, 2004). Usually, leisure time includes physical, mental, and social activities (Kåreholt et al., 2011), so it can be expected that they can relieve their stress during exercise. On the contrary, married individuals often prioritize their family, so choosing a type of exercise or exercise time is also expected to be significantly influenced by their partner or children. Also, the probability of overweight and obesity increases after marriage (Tang et al., 2024), and they married people usually exercise for less time than single people (Nomaguchi & Bianchi, 2004). Thus, it seems like their purpose in exercising is only to maintain their health, not to relieve stress. In conclusion, three stress relief factors (physical, mental, and social) seemed to show differences due to these contradictory features between single and married people.

Second, contrary to stress relief, the married males (group 3) and married females (group 4) showed significantly higher mean scores than the single groups in life satisfaction. According to Vernon (2010), marriage has many benefits, which include the advantages of finance and time. For example, married people can save money because they share a house, car, and meals with their partner. Also, they can distribute their time effectively, such as when one person is focused on work, another one can be focused on their children. In addition, as people progress from middle age to old age, a significant problem of life satisfaction is loneliness, and Oh et al.’s (2022) study found that single people’s life satisfaction decreased over 10 years. Additionally, marriage also has advantages such as a high life satisfaction when in a good marriage, which helps to reduce the death rate regardless of age (Ma & Gu, 2023). For these reasons, married people may report higher levels of life satisfaction compared to single individuals.

Finally, the married males (group 3) and married females (group 4) also showed higher mean scores than single groups in quality of life. Diverse factors, which include personal physical and psychological health, environments, social relationships, etc., are linked to people’s quality of life (Akranavičiūtė & Ruževičius, 2007). For example, high-income, higher-educated, and employed people show a higher quality of life than low-income, lower-educated, and unemployed people (Purba et al., 2021). However, in this study, all of the participants are equal members of society and participate in exercise, so it can be expected that marriage status was a significant factor in these results. According to G. E. Kim and Kim’s (2020) study, the authors found that single mothers’ quality of life was lower than that of married mothers because single mothers’ residential instability, higher stress levels, and depressive symptoms reduced their quality of life. Thus, marriage-related advantages such as financial and residential stability and emotional security may be associated with higher quality of life scores among married participants.

Regarding the relationship between physical exercise and stress relief, life satisfaction, or quality of life, it is difficult to draw a definitive conclusion about whether this relationship is inherently positive or negative, as individuals’ motivations for exercising vary. For instance, some individuals engage in exercise because they have a positive relationship with their bodies and find joy in physical activity, while others may exercise due to dissatisfaction with their bodies or as a means to escape from family-related stress. Moreover, the role of marital status in moderating this relationship remains unclear. The psychological benefits of exercise may differ between married and unmarried individuals. For example, married individuals might engage in exercise as a strategy to balance work and family responsibilities, whereas unmarried individuals might use exercise as a form of socialization or personal development. Additionally, age could act as a confounding variable, influencing both exercise habits and perceptions of life satisfaction.

It is important to note that this study does not directly assess the effects of exercise itself, as all of the participants were already engaged in physical activity. Instead, the study focuses on whether married and unmarried males and females perceive the effects of their exercise differently. This difference in perception may be influenced by various factors, including the intensity of their exercise, the personal significance of physical activity in their life stage (e.g., maintaining an attractive appearance may be more important for unmarried individuals), or ceiling effects, where individuals who are already highly satisfied with their lives may experience limited additional benefits from exercise.

Moreover, it is important to consider that the benefits commonly associated with marriage may largely depend on the quality of the marital relationship itself. Prior research has shown that individuals in high-conflict or unsatisfying marriages may experience lower life satisfaction and greater psychological distress than their single or divorced counterparts (Whisman et al., 2000; Williams & Umberson, 2004). Therefore, the observed differences in perceived well-being between married and unmarried participants should be interpreted with caution, as marital status alone may not fully capture the complexities of these experiences.

## 5. Conclusions and Future Research

This study investigated and compared stress relief, life satisfaction, and quality of life based on gender and marriage status among the members of society participating in the exercise. First, regardless of gender, the single participants showed higher mean scores in all sub-variables of stress relief (physical, social, and mental) than the married groups. These results seem reasonable because single people can more freely relieve stress by exercising without restrictions on time and place compared with married people. Next, also regardless of gender, the life satisfaction and quality of life mean scores were higher in the married groups than in the single groups. Unlike temporary stress relief, marriage-related factors such as reduced loneliness, emotional comfort, and financial or residential stability may be associated with higher life satisfaction and quality of life.

The results of this study showed differences based on the respective single and married groups’ characteristics. In the case of single people, there are benefits such as guaranteed autonomy and free time, but they can be sensitive to loneliness and anxiety. Conversely, married people can feel overwhelmed by caring for their children and partners, but, sometimes, the family becomes the most stable place or source of support for them. Given that this study only includes individuals who regularly engage in exercise, the results do not allow for a direct assessment of whether physical activity itself has a positive association with stress relief, life satisfaction, and quality of life. Rather, this study highlights differences in how married and unmarried men and women perceive the impact of their exercise. This suggests that the role of physical activity in psychological well-being may be shaped not only by exercise itself but also by life circumstances, personal motivations, and pre-existing levels of happiness. In conclusion, this study’s results showed psychological factor differences among members of society based on marriage status, but it is widely recognized that participation in exercise is positively related people’s mental health.

There are some limitations in this study, so the authors would like to suggest the following: First, this study only focused on the members of society participating in exercise. Therefore, future research needs to consider and compare exercise and non-exercise groups. Second, although this study revealed differences between single and married individuals, it did not account for potential confounding variables such as income, education level, or employment status. These socioeconomic factors are known to independently influence well-being and may partially explain the observed differences. Future research should statistically control for such variables using regression models or structural equation modeling (SEM) to better determine whether the differences are attributable to marital status itself. Third, the current study focused on a binary classification of marital status (single and married people), which may oversimplify the diversity of modern relationships. Future studies should expand their scope to include various relationship configurations, such as cohabiting couples, long-term single individuals, and divorced or widowed people, to gain a better understanding of how different relational contexts influence well-being. Fourth, this study used different Likert scale formats (five-point vs. seven-point) across measurement tools. Although these were retained to preserve the validity and comparability of each scale with prior research, this difference may affect direct comparisons between constructs. Therefore, future research should consider using standardized scale formats across measures to enhance comparability and reduce potential bias.

## Figures and Tables

**Table 1:** Descriptive statistics of survey respondents.

		Group 1 Single Males	Group 2 Single Females	Group 3 Married Males	Group 4 Married Females
Sex	Male	84 (100.0%)	-	76 (100.0%)	-
	Female	-	71 (100.0%)	-	80 (100.0%)
Age	20s	57 (67.9%)	53 (74.6%)	29 (38.2%)	30 (37.5%)
	30s	26 (31.0%)	17 (23.9%)	15 (19.7%)	20 (25.0%)
	40s	-	1 (1.4%)	10 (13.2%)	3 (3.8%)
	50s	1 (1.2%)	-	13 (17.1%)	17 (21.3%)
	Over 60s	-	-	9 (11.8%)	10 (12.5%)
Marital status	Single	84 (100.0%)	71 (100.0%)	-	-
	Married	-	-	76 (100.0%)	80 (100.0%)
Education	High school	8 (9.5%)	11 (15.5%)	5 (6.6%)	16 (20.0%)
	Bachelor	52 (61.9%)	32 (45.1%)	49 (64.5%)	47 (58.8%)
	Master	24 (28.6%)	25 (35.2%)	15 (19.7%)	13 (16.3%)
	Doctoral	-	3 (4.2%)	7 (9.2%)	4 (5.0%)
Employment status	Student	31 (36.9%)	31 (38.0%)	18 (23.7%)	24 (30.0%)
	Homemaker	-	-	2 (2.6%)	10 (12.5%)
	Salaried employment	22 (26.2%)	17 (23.9%)	16 (21.0%)	21 (26.3%)
	Self-employed	16 (19.0%)	15 (21.1%)	31 (40.8%)	14 (17.5%)
	Out of work	15 (17.9%)	12 (16.9%)	9 (11.8%)	11 (13.8%)
Frequency of participating in exercise (per week)	Less than once	5 (6.0%)	10 (14.1%)	4 (5.3%)	16 (20.0%)
	1–2 times	31 (36.9%)	33 (46.5%)	26 (34.2%)	21 (26.3%)
	3–4 times	28 (33.3%)	21 (29.6%)	24 (31.6%)	23 (28.7%)
	5–6 times	18 (21.4%)	6 (8.5%)	20 (26.3%)	15 (18.8%)
	7 times	2 (2.4%)	1 (1.4%)	2 (2.6%)	5 (6.3%)
	Total	84 (100.0%)	71 (100.0%)	76 (100.0%)	80 (100.0%)

**Table 2:** Results of exploratory factor analysis on stress relief and Cronbach’s alpha coefficient.

Items	1	2	3
(Mental)			
I feel good and relaxed after exercising.	**0.886**	0.176	0.213
I feel less tense or anxious through exercise.	**0.874**	0.188	0.183
I feel more confident through exercise.	**0.817**	0.167	0.208
I am able to control my emotions through exercise.	**0.814**	0.217	0.278
(Social)			
I have gotten along better with others through exercise.	0.135	**0.884**	0.147
I maintain good relationships with others through exercise.	0.174	**0.882**	0.192
I overcome difficult social situations through exercise.	0.261	**0.838**	0.210
I feel that exercise improves my social skills.	0.169	**0.834**	0.180
(Physical)			
I feel healthier through exercise.	0.276	0.202	**0.800**
I feel more physically comfortable through exercise.	0.042	0.109	**0.789**
I am able to get a deeper sleep through exercise.	0.359	0.210	**0.766**
I am pain-free through exercise.	0.371	0.277	**0.746**
Eigenvalues	6.212	1.852	1.317
Variance (%)	51.767	15.437	10.971
Cronbach’s alpha	0.920	0.920	0.865

**Table 3:** Results of exploratory factor analysis on life satisfaction and Cronbach’s alpha coefficient.

Items	1
I am living the life of my dream	0.910
I am satisfied with my life	0.904
If I could live my life over again, I would change almost noting	0.899
Overall, I live a happy life	0.813
Eigenvalues	3.313
Variance (%)	77.830
Cronbach’s alpha	0.894

**Table 4:** Results of exploratory factor analysis on quality of life and Cronbach’s alpha coefficient.

Items	1
Fun	0.938
Enjoyable	0.931
Valuable	0.926
Hopeful	0.916
Worthwhile	0.814
Eigenvalues	4.107
Variance (%)	82.148
Cronbach’s alpha	0.945

**Table 5:** Results of MANOVA by independent variables.

	*df*	*F*	*p*	*η* ^2^	post hoc	Mean Scores			
						Single Males	Single Females	Married Males	Married Females
Physical	3	32.452	<0.001 ***	0.241	a, b > c, d	3.94	4.03	3.10	3.15
Social	3	14.509	<0.001 ***	0.124	a, b > c, d	3.88	3.93	3.25	3.33
Mental	3	18.875	<0.001 ***	0.156	a, b > c, d	3.92	3.60	3.10	3.08
Life satisfaction	3	23.586	<0.001 ***	0.187	a, b < c, d	3.38	3.43	4.12	4.18
Quality of life	3	11.409	<0.001 ***	0.100	a, b < c, d	4.81	4.92	5.86	5.62

Note. *** *p* < 0.001, a = single males, b = single females, c = married males, d = married females.

**Table 6:** Results of the Scheffe post hoc analysis.

		Physical	Mental	Social	Life Satisfaction	Quality of Life
Single males	Single females	0.911	0.990	0.444	0.982	0.966
	Married males	<0.001 ***	<0.001 ***	<0.001 ***	<0.001 ***	<0.001 ***
	Married females	<0.001 ***	<0.001 ***	<0.001 ***	<0.001 ***	0.002 **
Single females	Single males	0.911	0.990	0.444	0.982	0.966
	Married males	<0.001 ***	<0.001 ***	<0.001 ***	<0.001 ***	<0.001 ***
	Married females	<0.001 ***	<0.001 ***	<0.001 ***	<0.001 ***	0.019 *
Married males	Single males	<0.001 ***	<0.001 ***	<0.001 ***	<0.001 ***	<0.001 ***
	Single females	<0.001 ***	<0.001 ***	<0.001 ***	<0.001 ***	<0.001 ***
	Married females	0.985	0.945	0.999	0.973	0.755
Married females	Single males	<0.001 ***	<0.001 ***	<0.001 ***	<0.001 ***	0.002 **
	Single females	<0.001 ***	<0.001 ***	<0.001 ***	<0.001 ***	0.019 *
	Married males	0.985	0.945	0.999	0.973	0.755

Note: *** *p* < 0.001, ** *p* < 0.01, * *p* < 0.05.

## Data Availability

The data presented in this study are available on request from the corresponding author.
